# A longitudinal analysis of posttraumatic growth and affective well-being among people living with HIV: The moderating role of received and provided social support

**DOI:** 10.1371/journal.pone.0201641

**Published:** 2018-08-06

**Authors:** Marcin Rzeszutek

**Affiliations:** Faculty of Psychology, University of Warsaw, Stawki, Warsaw, Poland; Boston University, UNITED STATES

## Abstract

**Objectives:**

The aim of this one-year longitudinal study was to examine the temporal relationship between the level of posttraumatic growth (PTG) and affective well-being, measured by the presence of positive and negative affect among people living with the HIV (PLWH). In addition, the moderating effects of received and provided support with respect to the above-mentioned relationship were investigated.

**Method:**

Study participants completed the following psychometric inventories: the Posttraumatic Growth Inventory (PTGI), the Positive and Negative Affect Schedule (PANAS-X), and the Berlin Social Support Scales (BSSS). Three assessments were performed: 129 patients were recruited for the first assessment, 106 patients agreed to participate in the second assessment, and 82 of the initial 129 participants (63.6%) participated in all three assessments.

**Results:**

An indirect association between PTG and positive affect was observed. However, no association was found between PTG and negative affect. Received support, but not provided support, completely moderated the relationship between PTG and positive affect.

**Conclusions:**

This study adds to the literature by examining the temporal relationship between PTG and affective-wellbeing among PLWH. It appears from the results that in this patient group, PTG may enhance the positive affect over time. However, receiving support is vital in this process.

## Introduction

Over the past two decades, especially after the advent of positive psychology in the early 2000s, several studies have been conducted on the positive consequences of traumatic events, referring to the phenomenon of posttraumatic growth (PTG) [1, 2, 3, 4, 5]. According to Tedeschi and Callhoun [4, 5], PTG occurs when an individual experiences highly challenging life events that manifest as profound transformations in several functional aspects of life such as improved social relationships, seeking of new life paths, greater appreciation of life, openness to spirituality, and awareness of personal strength. Several studies have been conducted on PTG, but many aspects of this positive phenomenon remain unclear [6, 7]. One of these is the association between PTG and psychological well-being (PWB), i.e. whether the above-mentioned positive changes after a traumatic experience improve the well-being of the trauma survivors over time. Further, if they do improve the well-being, what is the direction of this improvement [8]. According to Zoellner and Maercker [9], examining this research question is especially important for clinicians because if PTG is unrelated to PWB or other aspects of mental health, it remains only an interesting theoretical construct without practical clinical utility. The obvious hypothesis in this case would be that there is a positive association between these two variables. However, studies on this topic are very inconclusive. While some authors have found a positive link between PTG and PWB [10, 11, 12, 13], other studies indicate a lack of association [14, 15], negative association [16], or even a curvilinear relationship between PTG and PWB [17, 18]. These conflicting findings may be attributed to the multidimensional nature of PWB and its various operationalisations, in terms of the general quality of life, life satisfaction, or affective well-being [19, 20, 1]. Each of these dimensions may be differently related to PTG, precluding clear conclusions. Other authors noticed that the majority of studies were cross-sectional studies. Thus, these were unable to provide an understanding of whether PTG can accurately predict the improvement in the well-being domains [21]. Finally, in a meta-analytic review, Park [22] highlighted the role of various moderators (e.g., time passed after the trauma, social support received after the traumatic event) that should be considered for obtaining a detailed representation of the link between PTG and PWB.

The literature on HIV/AIDS is dominated by the negative consequences of HIV infection, which acts as a traumatic stressor and induces various mental disorders, including depression, anxiety, and posttraumatic stress disorder (PTSD) [23, 24, 25, 26, 27, 28, 29, 30]. In particular, HIV-related distress, manifested as depressive mood and negative affect may be constantly present among PLWH several years after HIV diagnosis [31] and is related to worse adherence to treatment [32] and faster HIV progression [33]. Conversely, research on positive aspects of living with HIV, including PTG, is relatively scarce [34, 35]. In particular, PTG in this patient group was related to higher viral load [36], less intense perceived HIV-related stigma [37], and better affective well-being [38]. In addition, the positive affect among PLWH predicted slower HIV progression [39], better adherence to treatment [40], fewer depressive symptoms [41], and lower mortality rate [42]. However, according to Sawyer et al. [43] the relationship between PTG and PWB among PLWH is unclear, and the central question is whether and how PTG in these patients may be associated with psychological advantages, especially considering that longitudinal studies on PTG among PLWH are scarce [44, 45], establishing only a few causal relationships.

There is considerable evidence showing a positive influence of received support on well-being [46, 47], especially on affective well-being [48]. On the other hand, some authors have reported a negative link between receiving support and PWB [49], in accordance with the equity theory [50]. The equity theory states that receiving support may intensify distress owing to the rule of reciprocity. Limited research has been conducted on the role of provided social support in PWB, but some studies [51, 52] have indicated that providing support may be more beneficial for PWB than receiving support, which is consistent with the esteem enhancement hypothesis [53]. With respect to PLWH, while the role of provided support remains largely unknown, several studies have shown a positive link between receiving support and good physical as well as mental functioning among PLWH [54, 55, 56]. By contrast, a relationship exists between a lack of support and exacerbation of HIV-related mental problems, especially depression [25, 57]. Furthermore, Rzeszutek et al. [45] observed a positive relationship between received support and PTG among PLWH, while Cieślak et al. [58] found that received support was positively associated only with the one PTG dimension, i.e. better relations with others. However, many studies that investigated the role of social support in PLWH are limited by several shortcomings, such as the lack of a clear definition and distinction between the different social support dimensions as well as the dominance of cross-sectional studies [59]. Therefore, I used a longitudinal study design and established a clear distinction between received and provided support to investigate the moderating effects of these social support dimensions on the link between PTG and affective well-being among PLWH.

## Current study

In this study, the link between the level of PTG and affective well-being, measured by the presence of positive and negative affect (PA/NA) was investigated in a one-year longitudinal study among PLWH. In addition, the moderating effects of received and provided support were explored for the above-mentioned relationship. The following hypotheses were formulated in line with longitudinal study design [60]:

1. There is a positive relationship between the level of PTG in the first assessment and the intensity of PA in the third assessment, while controlling for the level of PA in the first assessment.
2. There is a negative relationship between the level of PTG in the first assessment and the intensity of NA in the third assessment, while controlling for the level of NA in the first assessment.
3. Received support and provided support in the second assessment moderate the relationships described by the first and second hypothesis.

**Fig 1:**
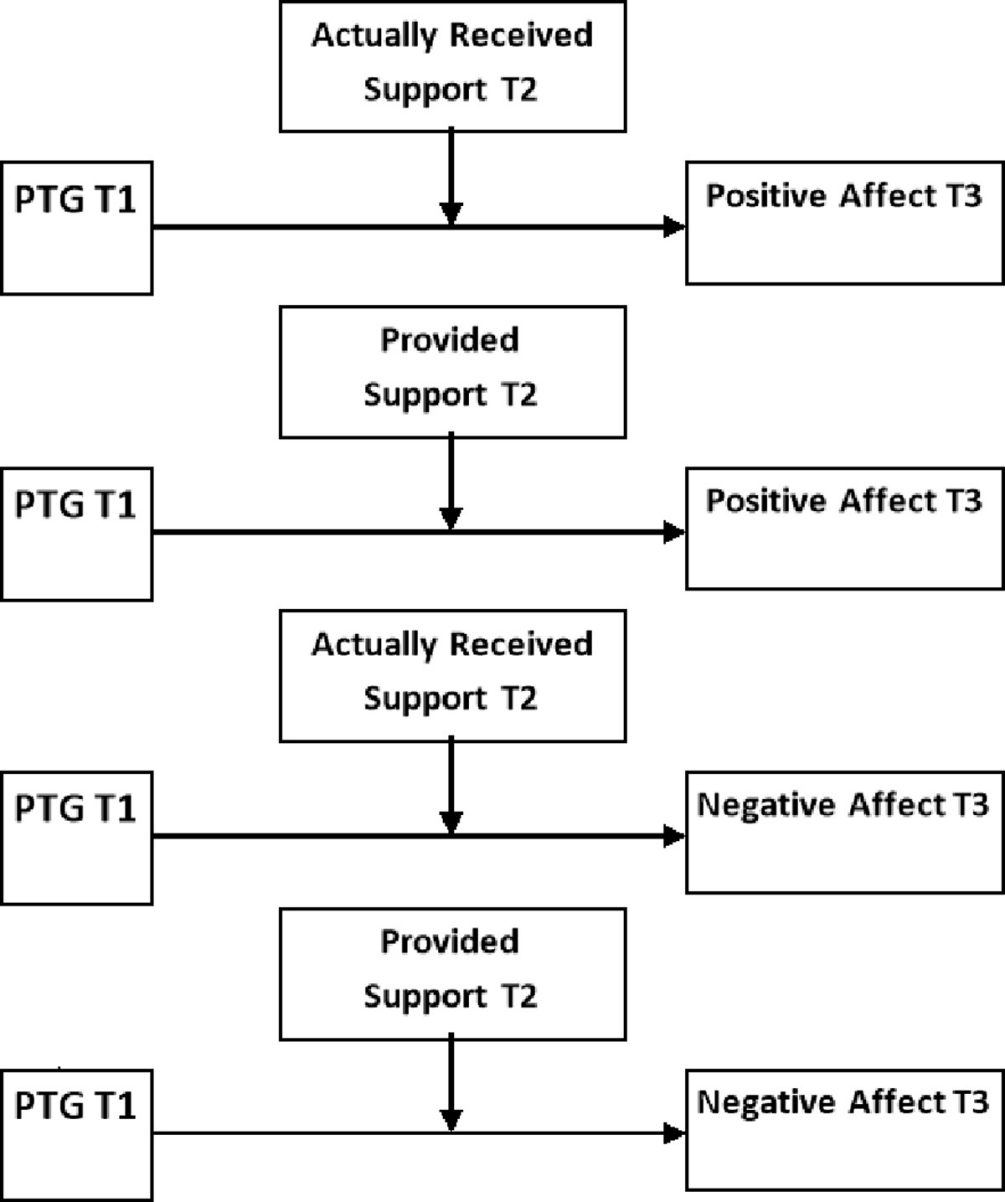
Preliminary hypothesised model. T1 –First Assessment; T2 –Second Assessment; T3 –Third Assessment.

A preliminary figure was designed to illustrate data analysis plan (Fig 1).

## Method

### Procedure

Patients admitted to the Hospital of Infectious Diseases in Warsaw were enrolled as study subjects. The subjects filled out a paper-and-pencil version of the inventories and participated in the study voluntarily because no remuneration for participation was provided. The study inclusion criteria encompassed being ≥ 18 years old, being medically diagnosed with HIV infection without other infectious co-morbidities (e.g. HCV) and undergoing treatment at aforementioned hospital. The exclusion criteria included HIV-related cognitive disorders that were identified by psychiatrists working at this hospital. The experimental design of this study was approved by the Senate Ethics Committee of the University of Finance and Management in Warsaw.

### Measures

To assess the intensity of PTG, a Polish adaptation of the Posttraumatic Growth Inventory [PTGI; 4] was used [61]. It should be noted that although the original PTGI comprises five specific domains of PTG (‘relating to others’, ‘new possibilities’, ‘personal strength’, ‘spiritual change’, and ‘appreciation of life’), the Polish adaptation of the PTGI included only four domains of PTG. Exploratory and confirmatory factor analyses revealed a four-factor structure for the PTG, including changes in self-perception (‘perceiving new possibilities, and ‘feeling of personal strength’), changes in relationships with others (‘feelings of greater connection with other people, increase in empathy, altruism’), greater appreciation for life (‘changes in life philosophy and current life goals, greater appreciation for every day’), and spiritual changes (‘better understanding of spiritual issues, increase in religiousness’). In the PTGI, participants were required to rate 21 positive statements that describe the various changes resulting from traumatic or highly challenging events that are provided at the beginning of the inventory. Study subjects were instructed to focus on their diagnosis of HIV infection as an example of a traumatic event. Statistical analyses are usually performed only for the global PTG score (sum of all items), as particular subscales in the Polish version of PTGI are highly intercorrelated [61]. In particular, Park and Helgeson [8] recommend unifactorial assessment of PTG, which represents a more valid method of measuring PTG compared to the analysis of the various dimensions of growth that may vary form one study to another. Cronbach’s α in the final sample population at the third assessment for the whole scale was α = .86, and for the four subscales, it varied from .81 to .85.

In order to assess affective well-being (the positive and negative affect), a Polish adaptation [62] of the PANAS-X was used [63]. The PANAS-X comprises 10 adjectives for positive affect (e.g., *proud*, *excited*, etc.) and 10 for negative affect (e.g., *frightened*, *hostile*, etc.). The participants were asked to evaluate their general affective states on a five-point response scale that ranged from 1 (not at all) to 5 (extremely). The Cronbach’s α coefficients in the studied final sample at the third assessment were .81 for the positive affect subscale and .83 for the negative affect subscale.

Social support was assessed using Schwarzer and Schulz’s [64] Berlin Social Support Scales (BSSS), adapted in Polish by Łuszczyńska et al. [65]. It evaluates a broad range of support dimensions. However, in this study, I used two scales: the actually received support and the provided support. The psychometric properties of the Polish version of the BSSS have been proven on various groups of patients, including those who had undergone bypass surgery or had experienced a heart attack as well as patients with chronic, degenerative spinal diseases [65]. These studies have confirmed the satisfactory reliability and validity of the tool. Cronbach’s α reliability coefficients in the final sample at the third assessment were .83 for received support and .85 for provided support.

The Table 1 clarifies the assessment plan, i.e., it summarizes which variables were assessed in the three consecutive assessments.

**Table 1:** Variables assessed in the three consecutive assessments.

	T1	T2	T3
Socio-medical Variables	x	x	x
PTG	x	-	-
Actually Received Support		x	-
Provided Support	-	x	-
Positive Affect	x	-	x
Negative affect	x	-	x

*Note*: x–The Variable Included In The Consecutive Assessment.

## Data analysis

Data analysis was conducted in three stages on the final sample of 82 participants with the use of IBM SPSS 24 statistical package [66]. Instead of using conventional statistical significance notation with p values, 95% confidence intervals were presented [67].

First, associations between all analysed interval variables and socio-medical data were investigated with the use of stepwise regression analysis in order to achieve more precise, unbiased means estimates when testing hypotheses and determining the main results of the study [67]. The stepwise regression was used only for exploring possible associations between analysed interval variables and socio-medical data and not for testing hypotheses.

Second, possible differences between three assessments were examined. Socio-medical data which were found to be related to interval psychological variables were used as covariates. Therefore, using the repeated measures analysis of covariance (ANCOVA), changes in the level of analysed variables over time were assessed. The statistical models included all the socio-medical data that were related to the interval psychological variables. Even if they were found to be related in only one stage of the study they were included in the model comparing the three assessments.

Finally, hierarchical regression analysis was performed to determine the main results of the study [67]. Four models were checked (Fig 1), where each time, the positive or negative affect in the third assessment was considered as the outcome variable, while the received or provided support from the second assessment was considered as the moderator of the relation between PTG in the first assessment and the outcome. Each model consisted of six blocks. In the first block, socio-demographical variables (sex, age, being in stable relationship, higher education and being employed) were analysed using the stepwise method. The first block was performed in order to control appropriate socio-demographical data. The stepwise method ensured the control of variables that were related to the explained variables, but it was not meant to test hypotheses. In the second block, clinical variables (CD4 counts, HIV duration, ART duration, and HIV/AIDS status) were analysed using also the stepwise method. The second block was performed in order to control for appropriate medical data. In the third block, the positive or negative affect (depending on which of these two was the outcome variable) in the first assessment was analysed using the entry method. The level of positive or negative affect in the first assessment was controlled. In the fourth block, the main effects of PTG in the first assessment as well as the received or provided social support (depending on which of these two was considered the moderator) in the second assessment were analysed using the entry method. The fifth and the last block assessed the interaction between PTG in the first assessment and the received or provided social support was analysed using the entry method. The interactions indicated moderation all other blocks were conducted in order to control for appropriate variables.

## Results

### Study sample

The first assessment was conducted between June 2016 and July 2016. Total of 200 patients with a clinical diagnosis of HIV infection were approached for the study. However, 44 patients refused to leave their contact details, and 27 patients did not indicate that HIV infection was a traumatic event for them. Thus, 129 patients met the inclusion criteria, i.e. they not only completed the questionnaires, but also agreed to provide their contact details (telephone number and/or e-mail address) to enable the researchers to contact them for the subsequent assessments, and indicated in the PTGI (see Measures) that the diagnosis of the HIV infection was traumatic for them. The second assessment was conducted between January 2017 and February 2017. Of the initial 129 participants, 106 agreed to participate in the second assessment. Finally, the last assessment was performed between May 2017 and June 2017, and 82 of the initial 129 participants (63.6%) participated in all three assessments. There were no missing data in the final data of the 82 participants. Participants who refused to participate in the follow-up assessments did not differ from the final sample population in terms of socio-medical variables and other studied variables. The Table 2 presents the socio-medical characteristics of the final study sample with 95% confidence intervals and interquartile ranges. The estimation was based on the National AIDS Centre Report data among officially declared PLWH being on antiretroviral treatment in Poland in 2017 [68].

**Table 2:** Socio-medical variables in the studied final sample (*N* = 82) with Confidence Intervals and interquartile ranges based on the national AIDS Centre Report data among officially declared PLWH being on antiretroviral treatment in Poland in 2017.

Variable	Final Sample (*N* = 82)
Sex	
Male	70 (85.4%, 76.4%÷94.4%)
Female	12 (14.6%, 5.6%÷23.6%)
Age in Years	
Range	21–76
(*M*±*SD*)	40.50 ±11.47 (*IR* = 12.25)
Relationship Status	
Stable Relationship	49 (59.8%, 48.8%÷70.8%)
Lack Of Stable Relationship	33 (40.2%, 29.2%÷51/2%)
Education	
Elementary	5 (6.1%, 0÷15.1%)
Secondary	26 (31.7%, 21.7%÷41.7)
University degree	51 (62.3%, 51.3%÷73.3%)
Employment	
Full employment	53 (64.6%, 53.6%÷75.6%)
Unemployment	23 (28.1%, 18.% ÷38.1%)
Retirement	6 (7.3%, 1.3%÷13.3%)
HIV/AIDS status	
HIV/AIDS status	
HIV+ only	66 (80.5%, 71.5%÷89.5%)
HIV/AIDS	16 (19.5%, 10.5%÷28.5%)
HIV Infection Duration in Years	
Range	1–30
(*M*±*SD*)	7.39±5.72 (*IR* = 7)
Antiretroviral Treatment (ART) Duration in Years	
Range	1–21
(*M*±*SD*)	5.76±4.88 (*IR* = 4)
CD4 Count	
Range	200–2000
(*M*±*SD*)	645.73 ±256.23 (*IR* = 342.50)

*Note*: *M* = Mean; *SD* = Standard Deviation; *IR*–interquartile range.

Table 3 presents socio-medical data, which were found to be related to psychological variables. The selection of socio-medical data was performed with the use of stepwise regression analysis.

**Table 3:** Socio-medical data associated with analysed psychological variables.

Variable	T1	T2	T3
PTG	Gender, *β* = .31 (.10÷.52)	Gender, *β* = .31 (.10÷.52)	-
Positive affect	-	-	Stable Relationship, *β* = -.22 (-.44÷-.01)
			CD4, *β* = .21 (.01÷.41)
Negative affect	CD4, *β* = -.28 (-.42÷-.01)	Employment, *β* = -.25 (-.47÷-.04)	Gender, *β* = .27 (.06÷.49)
Received Support	Employment, *β* = .22 (.01÷.44)	Higher Education, *β* = .33 (.13÷.52)	-
		Stable Relationship, *β* = -.31 (-.51÷-.11)	
Provided Support	-	Stable Relationship, *β* = -.34 (-.54÷-.13)	-
		Gender, *β* = .26 (.06÷.47)	
		Higher Education, *β* = .20 (.01÷.40)	

*Note*: *β*–Standardized Regression Coefficients with 95% Confidence Intervals; *T1* –First Assessment; *T2* –Second Assessment; *T3* –Third Assessment.

In the models concerning PTG at T1 and T2 participants’ gender was entered. In the model concerning positive affect in T3 stable relationship and CD4 were entered. Negative affect in T1 was found to be related to CD4 and negative affect in T2 was related to employment. There were relationships between received support and employment in T1 and between received support and higher education and stable relationship in T2. Provided support was related to stable relationship, participants’ gender and higher education in T2.

Table 4 presents the estimated marginal means for the analysed variables in three consecutive assessments obtained with the use of ANCOVA in which the socio-medical data mentioned in previous analysis were controlled along with the values of skewness and kurtosis. All the variables followed normal distribution. Repeated measures ANCOVA revealed no changes across the three assessments with respect to PTG, positive affect, negative affect, received support, or provided support.

**Table 4:** Estimated marginal means with 95% Confidence Intervals for PTG, positive and negative affect, received support and provided support for three assessments.

Analysed variable		Mean (*SE*)	
(Covariates)	*T1*	*T2*	*T3*
PTG	61.25(56.58÷65.91)	65.40 (60.16÷70.63)	63.52 (58.68÷68.34)
(Gender)	*S* = -.29(-.81÷.23); *K* = .53 (-1.47÷.59)	*S* = -.67(-1.19÷.15); *K* = .53 (-1.29÷.77)	*S* = -.33(-.85÷.20); *K* = .53 (-1.56÷.50)
Positive Affect	3.40(3.26÷3.56)	3.38(3.22÷3.57)	3.32(3.19÷3.48)
(CD4, Stable relationship)	*S* = -.42(-.94÷.10); *K* = .53 (-.93÷1.13)	*S* = -.11(-.64÷.41); *K* = .53 (-1.43÷.63)	*S* = .16(-.36÷.68); *K* = .53 (-1.75÷.31)
Negative Affect	2.24(2.05÷2.45)	2.18(1.99÷2.35)	2.22(2.03÷2.43)
(CD4, Employment, Gender)	*S* = .45(-.07÷.97); *K* = .53 (-1.93÷0.13)	*S* = .93(-.04÷1.45); *K* = .53 (-.27÷1.79)	*S* = .66(-.14÷1.18); *K* = .53 (-1.62÷.44)
Received support	29.47(27.30÷31.65)	31.58(29.39÷33.75)	31.84(29.60÷34.11)
(Employment, Higher education, Stable relationship)	*S* = -.67(-1.19÷.15); *K* = .53 (-1.42÷0.64)	*S* = -.74(-1.26÷.22); *K* = .53 (-1.14÷0.92)	*S* = -.68(-1.21÷.16); *K* = .53 (-1.22÷0.84)
Provided support	28.78(26.88÷30.70)	30.37(28.49÷32.67)	30.76(29.05÷32.49)
(Gender, Higher education, Stable relationship)	*S* = -.77(-1.09÷.05); *K* = .53 (-.09÷1.97)	*S* = -.56(-1.08÷.04); *K* = .53 (-1.31÷0.75)	*S* = -.61(-1.13÷.09); *K* = .53 (-.84÷1.22)

*Note*. *SE*–Standard Error; *T1* –First Assessment; *T2* –Second Assessment; *T3* –Third Assessment; *S*–Skewness with 95% Confidence Intervals; *K*–Kurtosis with 95% Confidence Intervals.

Table 5 presents the results of hierarchical regression analyses wherein PTG in the first assessment was analysed as a predictor, and positive or negative affect in the third assessment was analysed as the outcome, while received support in the second assessment was analysed as the moderator of the relationship between PTG in the first assessment and positive and negative affect in the third assessment. None of the clinical variables were related to PTG. Therefore, these were not included in the model. Regression coefficients of the health parameters in the second block are provided for reference. They were all excluded from the model.

**Table 5:** Results of multiple regression analysis. Received support as moderator of relation between PTG and positive affect and negative affect.

Dependent	Block	Predictor	Assessment	*β*	*ΔR*^*2*^
Positive affect	First	Stable Relationship	Third	-.22 (-.44÷-.01)	.05
	Second	CD4	Third	.01 (-.26÷.21)	-
		HIV Duration	Third	-.03 (-.36÷.55)	
		ARV Duration	Third	-.07 (-.54÷.34)	
		HIV/AIDS Status	Third	-.09 (-.36÷.13)	
	Third	Stable Relationship	Third	-.21 (-.40÷-.02)	.22
		+Positive Affect	First	.47 (.28÷.66)	
	Fourth	Stable Relationship	Third	-.15 (-.35÷.04)	.04
		Positive Affect	First	.42 (.20÷.61)	
		+PTG	First	.15 (-.06÷.34)	
		+ Actually Received Support	Second	.15 (-.07÷.34)	
	Fifth	Stable Relationship	Third	-.12 (-.36÷.03)	.04
		Positive Affect	First	.40 (.18÷.57)	
		PTG	First	.17 (-.03÷.37)	
		Actually Received Support	Second	.19 (-.07÷.33)	
		+PTG x Actually Received Support	First/Second	.20 (.01÷.38)	
Negative affect	First	Gender	First	.27 (.06÷.49)	.07
	Second	CD4	Third	.09 (-.17÷.30)	-
		HIV Duration	Third	.04 (-.26÷.64)	
		ARV Duration	Third	-.03 (-.54÷.34)	
		HIV/AIDS Status	Third	.07 (-.11÷.38)	
	Third	Gender	First	.25 (.04÷.47)	.02
		Negative Affect	First	.14 (-.07÷.36)	
	Fourth	Gender	First	.24 (.01÷.47)	.01
		Negative Affect	First	.15 (-.09÷.36)	
		PTG	First	.03 (-.19÷.27)	
		Actually Received Support	Second	.05 (-.29÷.15)	
	Fifth	Gender	First	.23 (-.01÷.48)	.01
		Negative Affect	First	.15 (-.09÷.36)	
		PTG	First	.03 (-.19÷.28)	
		Actually Received Support	Second	.04 (-.29÷.15)	
		PTG x Actually Received Support	First/Second	-.07 (-.20÷.23)	

*Note*:*β*–Standardized Regression Coefficients with 95% Confidence Intervals; *ΔR*^*2*^ –Change of the Variance Explained.

**Fig 2:**
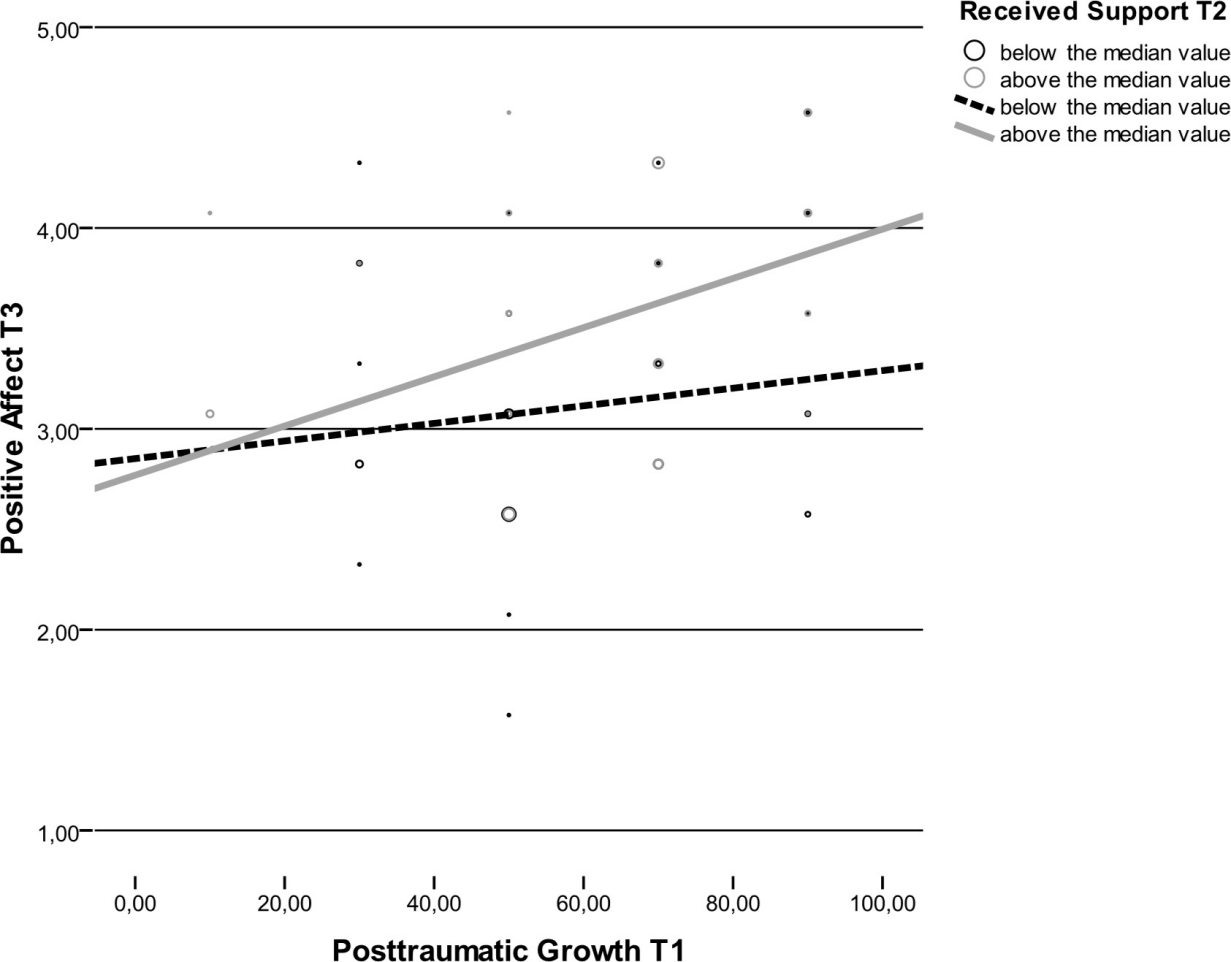
Scatterplot. Relation between posttraumatic growth in the first assessment and positive affect in the third assessment depending on the level of actually received support.

There was an interaction between received support in the second assessment and PTG level in the first assessment. Except for the control for the positive affect in the first assessment, all other predictors were not related to the explained variables. The meaning of interactions was determined using simple effects analyses [67]. Simple effects analyses based on the median split of received support (median [*Me*] = 47.00) were performed to find the meaning of the interaction. Regression analyses performed for the group of participants with received support below the median showed no relation between PTG in the first assessment and positive affect in the third assessment, *Beta* = .02 (-.32÷.25). The control for positive affect in the first assessment was the only predictor, *Beta* = .47 (.14÷.74). Regression analysis performed for the group of participants with received support above the median showed a relationship between PTG in the first assessment and positive affect in the third assessment, *Beta* = .31 (.12÷.70). The control for positive affect in the first assessment was also a predictor, *Beta* = .35 (.06÷.61) in the first assessment explained 9.2% of the variance in the positive affect in the third assessment. The higher the PTG level in the first assessment, the higher positive affect in the first assessment. However, this was true only for the group of participants whose level of received support was above the median (Fig 2).

There was no moderation effect on the received support in the second assessment of the relation between the PTG level in the first assessment and the negative affect in the third assessment.

Table 6 shows that there was no moderation effect of the relation between the PTG level in the first assessment and the positive affect in the third assessment on the provided support in the second assessment. There was also no moderation effect of the relation between the PTG level in the first assessment and the negative affect in the third assessment on the provided support in the second assessment. Participant’s sex was the only predictor. Women had higher levels of negative affect in the third assessment (*M* = 2.84; *SD* = .93) than men (*M* = 2.13; *SD* = .90).

**Table 6:** Results of multiple regression analysis. Provided support as moderator of relation between PTG and positive affect and negative affect.

Dependent	Block	Predictor	Assessment	*β*	*ΔR*^*2*^
Positive affect	First	Stable Relationship	Third	-.22 (-.44÷-.01)	.05
	Second	CD4	Third	.01 (-.26÷.21)	-
		HIV Duration	Third	-.03 (-.36÷.55)	
		ARV Duration	Third	-.07 (-.54÷.34)	
		HIV/AIDS Status	Third	-.09 (-.36÷.13)	
	Third	Stable Relationship	Third	-.21 (-.40÷-.02)	.23
		Positive Affect	First	.47 (.28÷.67)	
	Fourth	Stable Relationship	Third	-.16 (-.38÷.03)	.03
		Positive Affect	First	.41 (.22÷.63)	
		PTG	First	.13 (-.06÷.34)	
		Provided Support	Second	.13 (-.17÷.27)	
	Fifth	Stable Relationship	Third	-.15 (-.39÷.03)	.01
		Positive Affect	First	.40 (.20÷.63)	
		PTG	First	.13 (-.06÷.35)	
		Provided Support	Second	.14 (-.17÷.27)	
		PTG x Provided Support	First/Second	.05 (-.19÷.24)	
Negative Affect	First	Gender	First	.27 (.06÷.49)	.07
	Second	CD4	Third	.09 (-.17÷.30)	-
		HIV Duration	Third	.04 (-.26÷.64)	
		ARV Duration	Third	-.03 (-.54÷34)	
		HIV/AIDS Status	Third	.07 (-.11÷.38)	
	Third	Gender	First	.25 (.04÷.47)	.02
		Negative Affect	First	.14 (-.07÷.36)	
	Fourth	Gender	First	.25 (.02÷.49)	.01
		Negative Affect	First	.15 (-.08÷.36)	
		PTG	First	.04 (-.19÷.28)	
		Provided Support	Second	-.04 (-.30÷.16)	
	Fifth	Gender	First	.26 (.01÷.48)	.01
		Negative Affect	First	.14 (-.08÷.36)	
		PTG	First	.05 (-.18÷.29)	
		Provided Support	Second	-.06 (-.30÷.16)	
		PTG x Provided Support	First/Second	-.07 (-.17÷.31)	

*Note*: *β*–Standardized Regression Coefficients with 95% Confidence Intervals; *ΔR*^*2*^ –Change of the Variance Explained.

## Discussion

The results of this study were partially consistent with the first hypothesis because only an indirect association between PTG level and positive affect was observed. However, the second hypothesis was not positively verified because no relationship was found between the PTG level and negative affect. Thus, this study may provide an answer to important research question, i.e. whether the above-mentioned positive changes constituting PTG, which stems from HIV infection, are related to better well-being in this clinical sample over time. Several authors have shown that PTG is positively related to the emotional component of well-being (positive affect) [10, 69, 70, 71]. A previous trial also provides evidence that heightened left frontal brain activity, a common neurobiological mechanism, links PTG and positive affect [72]. Furthermore, Zoellner & Marcker [9] emphasize the need for a more detailed investigation of the role of positive emotions in the research on PTG. The need for further research on positive attributes, especially positive affect, has also been highlighted in contemporary HIV/AIDS literature [35, 73]. In particular, this result is in line with the observation of authors who have reported that PTG may have an indirect positive effect on PWB because this relationship is moderated by other variables [22, 1]. In particular, according to McAdams [74] and Triplet et al. [75], the indirect impact of PTG on PWB may be understood by search for a new perceptions and direction of life after trauma, resulting in subsequent changes in self-perception and the attitude towards other people. Nevertheless, the lack of association between PTG level and negative affect was surprising because several authors reported an association between the PTG level and a lower negative affect [10, 69, 76]. However, according to Friedrickson [77], positive and negative affects should not be treated as two ends of a unitary spectrum, but can constitute two separate constructs with different physiological backgrounds. This corresponds with other authors pointing that PTG is only associated with positive affect [78, 79].

The results of this study were partially consistent with the third hypothesis because received support, but not provided support, completely moderated the aforementioned relationship between PTG and positive affect only. Of the four analysed models, only the one that included the received support and positive affect, revealed moderation effects. This indicates that the PTG level at baseline was positively related to the intensity of positive affect in the third assessment, but this held true only for the participants who meanwhile received higher level of support. From the broader perspective, this finding is consistent with the social exchange theory, according to which received support is associated with improved well-being because individuals seek to maximize gains (receiving support from other people) and minimize losses (using up resources while supporting others) [80]. The positive association between received support and PWB has been reported by several studies [46, 47, 81]. With respect to PLWH, literature on HIV/AIDS shows several examples on how receiving social support improves PLWH’s affective well-being [82, 83], promotes health behaviours [84], protects from HIV-related stigma [85] or facilitates more adaptive coping strategies [55]. It is possible that for some PLWH, experiencing PTG could be a stimulus for seeking social support, given that this patient group still encounters several challenges in seeking and receiving support due to the stigma attached to HIV diagnosis [86, 87, 88, 89]. This is in compliance with the findings of Zeligman et al. [90] who not only observed a positive association between social support and PTG, but also found that PLWH who scored high on the PTGI reported lower levels of HIV-related stigma. A contradictory association between the intensity of PTG and HIV-related stigma has also been reported by Murphy and Hevey [37]. It is noteworthy that the current study did not provide evidence for the role of provided support in the link between PTG and PWB among PLWH. Although some research projects [51, 52] have indicated that providing support may be more beneficial for PWB than receiving support, other studies have highlighted the emotional costs of providing social support [91, 92], including the cost for HIV/AIDS care providers [93], which is in line with the aforementioned social exchange theory. In summary, the role of provided support among PLWH remains unclear. However, this null finding may be interpreted in the context of the aforementioned challenges that PLWH face during the process of seeking, receiving, and perhaps providing support [87].

### Strengths and limitations

This longitudinal study is theory-driven wherein three assessments were performed for the study variables, which are the strengths of this study. Nevertheless, the limitations also need to be acknowledged. First, the study had a relatively high dropout rate, resulting in a comparatively low final sample size at the third assessment. Specifically, low final sample size did not permit to assess the effect size of the studies associations with high accuracy. This is why the range of confidence intervals is so vast. In addition, due to organisational reasons, the study sample was diverse with respect to the duration of HIV infection (although this clinical variable was not a related to the explained variable) and consists of highly functional PLWH, with a good control of HIV infection (see CD4 count). Future studies should focus on a more homogenous HIV-infected sample when it comes to HIV infection duration, as well as on a more heterogeneous sample with respect to viral suppression. Furthermore, some authors criticise the PTGI as a retrospective measurement of growth [6], possibly impeding a detailed assessment of growth in case of physical illness [94].

### Conclusions

This study adds to the literature by examining the temporal relationship between PTG and affective well-being among PLWH. It appears that in this patient group, PTG may be positively related to positive affect over time. However, received support is crucial for this process. Research on HIV/AIDS as well as HIV counselling should concentrate more on the promotion of positive attributes in this patient group, as emphasized in contemporary literature [35].

## Supporting information

S1 Dataset (SAV)
